# Genomic predictions combining SNP markers and copy number variations in Nellore cattle

**DOI:** 10.1186/s12864-018-4787-6

**Published:** 2018-06-05

**Authors:** El Hamidi A. Hay, Yuri T. Utsunomiya, Lingyang Xu, Yang Zhou, Haroldo H. R. Neves, Roberto Carvalheiro, Derek M. Bickhart, Li Ma, Jose Fernando Garcia, George E. Liu

**Affiliations:** 1 USDA Agricultural Research Service, Fort Keogh Livestock and Range Research Laboratory, Miles City, MT 59301 USA; 2 Departamento de Medicina Veterinária Preventiva e Reprodução Animal, Faculdade de Ciências Agrárias e Veterinárias, UNESP - Univ Estadual Paulista, Jaboticabal, SP 14884-900 Brazil; 3 Institute of Animal Science, Chinese Academy of Agricultural Science, Beijing, 100193 China; 4 College of Animal Science and Technology, Northwest A&F University, Shaanxi Key Laboratory of Agricultural Molecular Biology, Yangling, Shaanxi 712100 China; 5 Departamento de Zootecnia, Faculdade de Ciências Agrárias e Veterinárias, UNESP - Univ Estadual Paulista, Jaboticabal, SP 14884-900 Brazil; 6 Animal Genomics and Improvement Laboratory, BARC, USDA-ARS, Beltsville, MD 20705 USA; 7 Department of Animal and Avian Sciences, University of Maryland, College Park, MD 20742 USA; 8 Departamento de Apoio, Produção e Saúde Animal, Faculdade de Medicina Veterinária de Araçatuba, UNESP – Univ Estadual Paulista, Araçatuba, SP 16050-680 Brazil

**Keywords:** Genomic selection, Complex trait, CNV, SNP, Nellore cattle

## Abstract

**Background:**

Due to the advancement in high throughput technology, single nucleotide polymorphism (SNP) is routinely being incorporated along with phenotypic information into genetic evaluation. However, this approach often cannot achieve high accuracy for some complex traits. It is possible that SNP markers are not sufficient to predict these traits due to the missing heritability caused by other genetic variations such as microsatellite and copy number variation (CNV), which have been shown to affect disease and complex traits in humans and other species.

**Results:**

In this study, CNVs were included in a SNP based genomic selection framework. A Nellore cattle dataset consisting of 2230 animals genotyped on BovineHD SNP array was used, and 9 weight and carcass traits were analyzed. A total of six models were implemented and compared based on their prediction accuracy. For comparison, three models including only SNPs were implemented: 1) BayesA model, 2) Bayesian mixture model (BayesB), and 3) a GBLUP model without polygenic effects. The other three models incorporating both SNP and CNV included 4) a Bayesian model similar to BayesA (BayesA+CNV), 5) a Bayesian mixture model (BayesB+CNV), and 6) GBLUP with CNVs modeled as a covariable (GBLUP+CNV). Prediction accuracies were assessed based on Pearson’s correlation between de-regressed EBVs (dEBVs) and direct genomic values (DGVs) in the validation dataset. For BayesA, BayesB and GBLUP, accuracy ranged from 0.12 to 0.62 across the nine traits. A minimal increase in prediction accuracy for some traits was noticed when including CNVs in the model (BayesA+CNV, BayesB+CNV, GBLUP+CNV).

**Conclusions:**

This study presents the first genomic prediction study integrating CNVs and SNPs in livestock. Combining CNV and SNP marker information proved to be beneficial for genomic prediction of some traits in Nellore cattle.

**Electronic supplementary material:**

The online version of this article (10.1186/s12864-018-4787-6) contains supplementary material, which is available to authorized users.

## Background

Genomic prediction is the estimation of breeding values using genetic variations such as single nucleotide polymorphism (SNP) [1]. Ideally, breeding values would be predicted as the sum of the effects of all inherited quantitative trait nucleotides (QTNs). As QTNs are not known in practice, genome-wide SNP markers have been proposed as surrogates to indirectly capture the effects of causal variants [1, 2]. However, due to incomplete linkage disequilibrium (LD) with other variants [3–7], SNP markers may fail to capture all the effects of variants causing missing heritability or phenotypic deviations, thus genomic estimated breeding values (GEBV) based on SNPs may represent only a component of the true breeding value (TBV) [8]. Missing heritability was defined as the proportion of genetic variation not accounted for by SNPs but predicted to be present due to heritability. Another possibility is that genetic effects are not due to the common SNPs, but due to other kinds of genetic variants, such as microsatelites and copy number variations (CNV) [9, 10].

In the last ten years, attention has been drawn to CNVs, as they are deemed to impact phenotypes. CNVs are structural variations larger than 50 bp in the form of insertions, deletions, duplications, inversions and translocations [11, 12]. For example, a number of studies indicate chromosomal translocations and subsequent duplications of the KIT gene are involved in several distinct cattle coat phenotypes [13, 14], suggesting that the different modifications of the KIT gene can influence coat color in cattle [14].

Given the ubiquity of immunity related genes that coincide with CNVs, there are likely many more immunity traits that are influenced by CNVs. Antimicrobial peptides (AMPs) represent a class of copy number variable genes within livestock species that function as part of the innate immune response to pathogens. The β-defensin class of AMPs appears to be copy number variable in several livestock species, but most notably in cattle [15, 16]. The lingual antimicrobial peptide (LAP) and tracheal antimicrobial peptide (TAP) genes share a high degree of sequence homology with β-defensins, but AMPs are exclusive to cattle [17]. Additionally, the BSP30A gene, which is an important salivary AMP, was found to be highly copy number variable within cattle of different breeds [16]. Finally, cathelicidin-type AMPs such as CATHL4 [16] and PGN3 [18] have been identified as highly variable among pig and cattle individuals, respectively. MHC gene family members have been frequently found to be copy number variable in livestock species. A duplication of the CIITA gene, which encodes a trans-activator of the MHC class II receptor, was found in cattle that had resistance to ingested nematodes [19]. In addition, studies on the loss of copy number of MHC class II genes within other species have revealed increased susceptibility of that species to pathogens and cancers, such as the Tasmanian devil facial tumor epidemic [20]. This serves as a warning to all animal breeders, as a loss of diversity at this locus due to improperly managed selective breeding or imposed population bottlenecks could increase the susceptibility of their herds to epidemics [21]. Several other classes of immunity related gene families have also been identified as copy number variable in livestock species. Expansion and contraction of the workshop class I (WC1) gene family has been identified in cattle [15, 16]. WC1 genes are unique to the cattle, sheep, and pig genomes, and encode pattern recognition receptors expressed on γδ-T cells [22].

The two 1000 human genome structural variation (SV) papers reported multiple types of CNVs, including deletions, tandem duplications, novel sequence insertions and mobile element insertions [11, 23]. Mills et al. [11] studied four CNV formation mechanisms by examining the breakpoint junction sequence: (1) NAHR or non-allelic homologous recombination, associated with homologous sequence; (2) NH or “non-homologous” rearrangements without sequence similarity, including NHEJ (non-homologous end-joining) and (MMBIR) microhomology-mediated break-induced replication; (3) VNTR (the shrinking or expansion of variable number of tandem repeats, often involving simple sequences by slippage; and (4) MEI (mobile element insertions, including transposition and retrotransposition of common repeats). Within these CNV types, deletions are often mediated by NAHR and are the easiest ones to detect, genotype and validate. Therefore, deletions were extensively studied by [11]; 2/3 of reported events were deletions and almost all (98.8%) validations were on deletions. Deletion’s unique advantages are that their locations and allele types are well defined and easy to assess. For a single deletion, its location is restricted to the allele’s locus and can be easily derived. Its alleles normally can only be one of these three types: no deletion (0,0), heterozygous deletion (− 1,0) and homozygous deletions (− 1,-1).

By comparing deletion genotypes with genotypes of nearby SNP, Mills et al. [11] found, consistent with earlier studies [3–7], that 81% of common deletions had one or more SNPs with which they are strongly correlated. This suggests that many deletions mapped will be identifiable through tagging SNPs. However, a fifth of the genotyped deletions were not tagged by HapMap SNP, implying that these CNV should be genotyped directly. In our cattle study [7], we observed a similar result, i.e. 75% simple deletions displayed LD with SNPs while the remaining 25% did not, suggesting that these events are not tagged by the BovineHD SNPs. Similarly, Handsaker et al. [24] used whole genome sequence data to detect and impute CNV and found that most of common deletions and biallelic duplications were well imputed whereas the imputation accuracy for common multi-allelic CNV or mCNV, especially duplications with three or more segregating alleles was lower [24]. Additionally, the LD properties of complex SVs (e.g., mCNV like tandem duplications or novel sequence insertion) have not yet been fully ascertained because methods for genotyping such CNVs with high accuracy just emerged and was only reported and applicable for human data [24, 25].

CNV can function either as causal variants or as tagging markers. A human study found that CNVs captured around 18% of the total variation in gene expression in cultured lymphocytes [26]. Furthermore, studies revealed several CNVs with effects on livestock economically important traits such as milk production, residual feed intake in Holstein cows and disease resistance in Angus cattle [7, 27–29]. As CNVs have been shown to affect gene structure and dosage, they may have drastic effects on phenotypes, altering gene regulation and exposing recessive alleles [7, 28, 30–33]. Considering the critical role of CNVs in complex traits, genomic prediction integrating both SNP and CNV may offer novel insights for elucidating complex traits and understanding the missing heritability. However, in the last decade, nearly all genomics predication in farm animal were conducted based on only SNP using GBLUP and Bayesian methods. Up to now, there are no reports of the joint use of SNP and CNV genotypes in genomic prediction in livestock [1, 34, 35]. We recently published a CNV-based study of growth traits using high density SNP microarray data in *Bos indicus* cattle. We detected 17 CNVs significantly associated with seven growth traits [36]. The objectives of this study were to integrate CNV (deletions and biallelic duplications) with SNPs into genomic evaluation using GBLUP and Bayesian methods and investigate their impact on the genomic prediction accuracy.

## Methods

### Phenotypic data

Estimated breeding values (EBVs) were based on Best Linear Unbiased Predictor (BLUP) estimates of single-trait animal models obtained from routine genetic evaluations using performance and pedigree data from the database (available at: http://www.gensys.com.br/home/show_page.php?id=701). Phenotypes used to fit the models comprised records from 542,918 animals born between 1985 and 2011, and raised in 243 grazing-based herds. The evaluated traits included birth weight (BW), post weaning gain (PWG), weaning gain (WG), carcass conformation at weaning (CW), muscling at weaning (MW), carcass finishing precocity at weaning (PW), carcass conformation at yearling (CY), muscling at yearling (MY) and carcass finishing precocity at yearling (PY). Conformation, finishing precocity and muscling traits (CPM) were based on recorded visual scores assigned in a discrete ordered scale, relative to the animals of the same management group (for a more detailed description of the traits, see Neves et al. 2014 [37]). For each trait, only EBVs of animals whose accuracy (i.e., square root of reliability, calculated based on prediction error variance estimates) was > 0.50 were analyzed. The number of animals used in the study and heritability of traits analyzed are presented in Table 1.

**Table 1:** Number of animals and heritabilities of traits analyzed

Trait	N	h^2^
BW	2058	0.37
CW	2032	0.25
CY	1979	0.31
MW	2032	0.26
MY	1979	0.30
PW	1982	0.25
PWG	1990	0.33
PY	1979	0.31
WG	2052	0.26

In this study, genomic prediction analysis was carried out using de-regressed EBV (dEBVs) instead of EBVs as the response variable in order to remove any bias due to double counting phenotypic and pedigree information. De-regressing of EBVs was performed according to the approach proposed by [38] which removed parent average (PA) effects and also accounted for heterogeneous variances. To test the performance of the proposed models, the dataset was randomly split into two datasets, 2/3 and 1/3 of the data for training and validation, respectively and the analysis was replicated five times.

### SNP genotyping and quality control

A total of 2230 Nellore animals (*Bos indicus*) were genotyped for 777,962 SNP markers with the Illumina BovineHD BeadChip assay. This data builds on previously published studies [37, 39]. The quality control step consisted of excluding SNP markers with minor allele frequency less than 0.02 and SNPs with Call Rate (CR_SNP_) < 0.98 and Fisher’s exact test *P*-value for Hardy-Weinberg Equilibrium (HWE) < 1 × 10^− 5^.

### CNV segmentation and genotyping

The multivariate CNV calling approach of Golden Helix SVS 8.3.0 (Golden Helix Inc., Bozeman, MT, USA) was used to detect common CNV events. This is because other traditional CNV discovery methods are not designed to find common CNVs but to report more CNVs [7]. In total, 992,350 CNVs were detected, as described previously [36]. By merging all the segments, 445 non-redundant CNV events were identified in the 2230 samples. After filtering away CNVs over 5 Mb and CNVs with frequency < 0.45% (i.e. appearing in less than 10 samples), a total of 231 CNVs with high confidence, ranging from 894 bp to 4,855,088 bp, were retained and used in further analysis.

After visual inspection of the histograms of segment mean intensities (LRR), all 231 CNVs were assigned into 2 categories: CNV events with simple and distinct genotype clusters or CNV with multiallelic and complex genotype clusters. Deletions and biallelic duplications can be genotyped if the clusters representing different genotypes are sufficiently distinct. Based on this classification and event frequency, three different CNV subsets were tested and used in genomic prediction analyses: 1) common deletions (*n* = 55) with frequency > 5%; 2) all deletions (*n* = 72) and 3) all deletions and biallelic duplication (*n* = 173) (Additional file 1: Table S1).

### Statistical analysis

The first three models used to estimate DGVs considering SNP effects only are the following: 1) Bayesian regression model (BayesA), a mixture Bayesian model (Bayes B) and a GBLUP model without polygenic effects (GBLUP). All three models accounted for additive effects only.

The first approach to combine SNP marker information and CNV information (BayesA+CNV) is described below. In this approach we assume that SNP effects and CNV effects contribute to the genetic variance. Using this approach the effects of variants will be modeled as the following:

1 $$ {y}_i=\mu +\sum \limits_{k=1}^p{x}_{ik}{b}_k+\sum \limits_{l=1}^m{z}_{il}{g}_l+{e}_i $$

Where *y*_*i*_ is the pseudo-phenotype (dEBV) for animal i, *μ* is the overall mean, *x*_*ik*_ is the SNP marker genotype for animal *i* at locus *k* (*k* = 1, 2…, *p*)coded as the number of copies of minor allele, *b*_*k*_ is the *k*^*th*^ SNP effect, *z*_*il*_ is the CNV genotype for animal *i*, *g*_*l*_ is the *l*^*th*^ CNV effect and *e*_*i*_ is the residual term.

For CNV effects, a flat prior was assumed since the number of CNVs is several folds smaller than the number of observations therefore allowing the data to drive the inferences of CNV effects.

The second approach to incorporate SNP markers and CNVs is a mixture model (BayesB+CNV) similar to the model in eq. (1) except for the SNP effects part $\sum \limits_{k=1}^p{x}_{ik}{b}_k{I}_k$ where *x*_*ik*_ is the genotype of the *k*^*th*^ marker, coded as the number of copies of the minor allele, *b*_*k*_ is the effect of marker *k*, and *I*_*k*_ is an indicator variable that is equal to 1 if the *k*^*th*^ marker has a non-zero effect on the trait and 0 otherwise. A binominal distribution with known probability π = 0.01 was assumed for *I*_*k*_. As opposed to SNPs, a mixture distribution was not assumed for CNVs, since the number of CNVs is small.

A third approach is GBLUP where CNVs were modeled as a covariate which can be described as:

2 $$ \boldsymbol{y}=\boldsymbol{Xb}+\boldsymbol{Za}+\boldsymbol{e} $$

where **y** is the vector of dEBVs, **b** is a vector of fixed CNV covariates coded as − 1, 0, 1 for neutral, loss and gain states respectively, **a** is the vector of random animal additive effects and **e** is the vector of residual terms. The direct genomic value (DGV) was calculated as:

3 $$ \boldsymbol{DGV}=\boldsymbol{X}\widehat{\boldsymbol{b}}+\boldsymbol{Z}\widehat{\boldsymbol{u}}\kern0.5em $$

Where **DGV** is the vector of direct genomic values, **X** is the matrix of CNV covariates, **b** is the vector of CNV effects, **Z** is the matrix of genotypes and **u** is the vector of estimated SNP effects.

Models adopted in this study were compared using the following criteria: Pearson’s correlation between dEBV and DGV, mean squared error of Prediction (MSE) and the regression slope of dEBVs on DGVs for animals in the validation dataset in order to test the inflation/deflation degree of genomic predictions.

Gibbs sampler was used with a chain of 90,000 iterations for each parameter, with a burn-in period of 10,000 iterations and a sampling interval of 100 iterations. Convergence testing was performed for all parameters including SNP effects following Geweke’s (1992) [40] and Heidelberger and Welch’s (1983) [41], and visual analysis of trace plots was also performed using Bayesian Output Analysis program.

## Results and discussion

### CNV detection

Out of 231 CNV, 95 (41.13%) were pure deletions. Within the remaining CNVs, only 12 CNVs (5.19%) have duplication frequency > 5% and all other 124 CNV had duplication frequency < 5%. Based on CNV classification and event frequency (Methods), three different CNV subsets were tested and used in genomic prediction analyses: 1) common deletions (*n* = 55) with frequency > 5%; 2) all deletions (*n* = 72) and 3) all deletions and biallelic duplication (*n* = 173) (Additional file 1: Table S1).

As we described previously [42], most of deletions reported in this study by the SVS’s multivariate option were either no deletions or homozygous deletions, with only a handful of events were heterozygous deletions. A similar observation of two alleles was found for biallelic duplications, the event was either with no duplication or with duplication. These results indicated that deletions and biallelic duplications could be accurately genotyped with defined genomic coordinates and mainly 2 states (with or without deletion or duplication), which were similar to the behaviors of common SNPs. As demonstrated for human and cattle CNVs previously [4, 36, 43], the assumed additive model was largely satisfied when deletions and biallelic duplications were included in genetic prediction.

### Genomic prediction

**Fig. 1:**
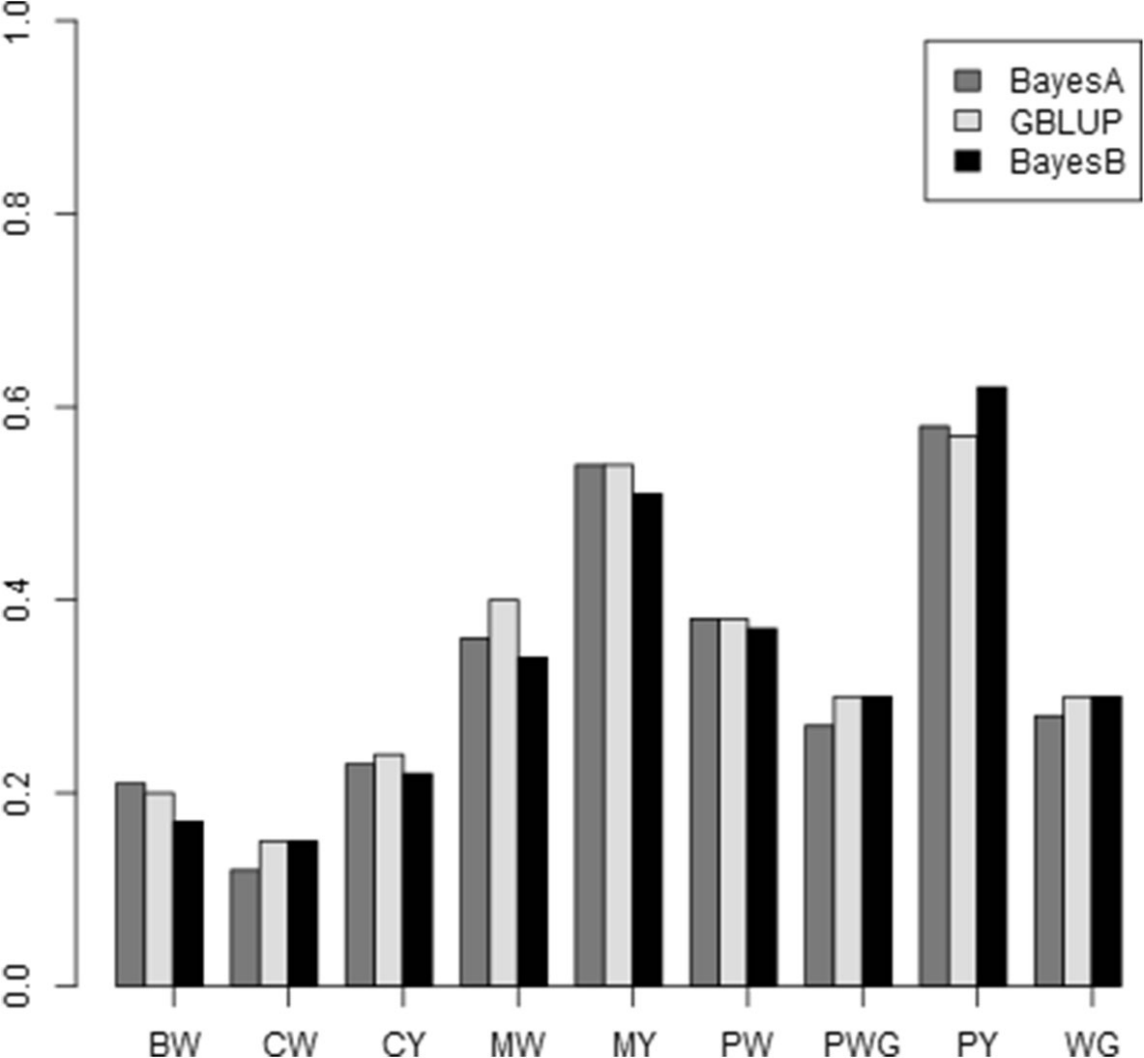
Prediction accuracies calculated as Pearson’s correlations between direct genomic values (DGVs) and dEBVs of animals in the validation data sets using BayesA, BayesB and GBLUP

Different methods of incorporating CNVs into genomic evaluation were compared based on their prediction accuracies. Using the average of 5 replicates, prediction accuracies computed as Pearson’s correlation between DGV and dEBV for all nine traits using SNP markers are shown in Table 2. The accuracy for BW trait was 0.21 using BayesA and dropped to 0.17 and 0.20 using BayesB and GBLUP respectively. For MW, higher accuracy was seen using GBLUP (0.40) compared to models BayesA and BayesB with accuracies of 0.36 and 0.34 respectively. The highest prediction accuracy was noticed for PY using BayesB model (0.62). On average GBLUP model resulted in slightly higher genomic prediction accuracies than BayesA and BayesB. The genomic prediction accuracy results of the three models differed from trait to trait as displayed in Fig. 1.

**Table 2:** Pearson’s correlations between dEBVs and DGVs of 9 traits for different models using SNP markers only and combining SNP and CNV information

	BayesA	BayesA+CNV			Bayes B	BayesB+CNV			GBLUP	GBLUP+CNV		
Trait	SNPs^a^	del^b^	All del^c^	All^d^	SNPs^a^	del^b^	All del^c^	All^d^	SNPs^a^	del^b^	All del^c^	All^d^
BW	0.21 (±0.03)	0.22 (±0.02)	0.21 (±0.04)	0.24 (±0.02)	0.17 (±0.02)	0.23 (±0.02)	0.23 (±0.03)	0.22 (±0.06)	0.20 (±0.03)	0.20 (±0.02)	0.20 (±0.02)	0.20 (±0.04)
CW	0.12 (±0.01)	0.10 (±0.01)	0.10 (±0.01)	0.10 (±0.03)	0.15 (±0.01)	0.15 (±0.04)	0.13 (±0.04)	0.14 (±0.02)	0.15 (±0.04)	0.16 (±0.01)	0.14 (±0.04)	0.14 (± 0.05)
CY	0.23 (±0.03)	0.23 (± 0.04)	0.22 (±0.03)	0.20 (±0.02)	0.22 (±0.03)	0.19 (±0.02)	0.19 (±0.05)	0.19 (±0.02)	0.24 (±0.01)	0.22 (±0.01)	0.21 (±0.02)	0.22 (± 0.03)
MW	0.36 (±0.01)	0.34 (± 0.01)	0.33 (±0.02)	0.36 (±0.01)	0.34 (±0.01)	0.39 (± 0.02)	0.38 (±0.03)	0.38 (±0.03)	0.40 (±0.02)	0.39 (±0.01)	0.39 (±0.04)	0.40 (± 0.04)
MY	0.54 (±0.05)	0.53 (± 0.06)	0.50 (±0.03)	0.53 (±0.04)	0.51 (±0.04)	0.56 (±0.02)	0.54 (±0.04)	0.54 (±0.04)	0.54 (±0.03)	0.52 (±0.04)	0.50 (±0.02)	0.55 (± 0.02)
PW	0.38 (±0.02)	0.36 (±0.03)	0.34 (±0.05)	0.36 (±0.01)	0.37 (±0.03)	0.38 (±0.01)	0.34 (±0.04)	0.40 (±0.02)	0.38 (±0.04)	0.36 (±0.03)	0.32 (±0.03)	0.36 (± 0.05)
PWG	0.27 (±0.02)	0.24 (±0.04)	0.23 (±0.03)	0.23 (±0.03)	0.30 (±0.01)	0.26 (±0.02)	0.26 (±0.03)	0.26 (±0.04)	0.30 (±0.01)	0.26 (±0.03)	0.24 (±0.02)	0.27 (± 0.02)
PY	0.58 (±0.04)	0.58 (±0.03)	0.58 (±0.04)	0.58 (±0.04)	0.62 (±0.02)	0.57 (±0.02)	0.56 (±0.03)	0.58 (±0.05)	0.57 (±0.03)	0.59 (±0.04)	0.59 (±0.04)	0.59 (± 0.04)
WG	0.28 (±0.05)	0.22 (±0.04)	0.20 (±0.03)	0.21 (±0.05)	0.30 (±0.03)	0.21 (±0.03)	0.20 (±0.03)	0.22 (±0.06)	0.30 (±0.01)	0.22 (±0.04)	0.22 (±0.05)	0.22 (± 0.03)

^a^Only SNPs

^b^SNPs and only common deletions with frequency greater than 5% were included in the model (55 CNVs)

^c^SNPs and all deletions were included in the model (72 CNVs)

^d^SNPs and all deletions and biallelic duplications (173 CNVs)

The prediction accuracies integrating CNVs are also presented in Table 2. Three different CNV subsets were tested (common deletions with frequency greater than 5%, all deletions, and all deletions and biallelic duplications). A small increase in prediction accuracy was seen for BW for all models across all three scenarios. The highest increase was noticed for model BayesB+CNV using all deletions (0.23 vs. 0.17). Further, prediction accuracy slightly increased for MW, MY and PW traits. Using BayesB model, the prediction accuracy for MW was 0.34, the accuracy increased when including CNVs (BayesB+CNV) to 0.39, 0.38 and 0.38 for common deletions greater than 5%, all deletions, and all deletions and biallelic duplications respectively.

A decrease in accuracy was also noticed when incorporating CNVs in the prediction. Accuracy for trait CW decreased from to 0.12 using BayesA to 0.10 using BayesA+CNV. The largest decrease in prediction accuracy was seen for WG using BayesB+CNV model. This decrease could be due to redundant information of CNVs already captured by SNP markers. On average, using common deletion CNVs with frequency greater than 5% resulted in higher accuracies. Furthermore, GBLUP+CNV slightly outperformed BayesA+CNV and BayesB+CNV. This gain in accuracy was observed when including CNVs into GBLUP type of approach. A plausible explanation to the behavior seen using this approach is that the genomic relationship matrix G used in GBLUP does not capture all the genetic variation, therefore including CNVs as covariates may explain part of the missing genetic variance and thus improving the prediction accuracy.

By evaluating the models in this study using the MSE criterion (Table 3), we found that the goodness-of-fit of the model did not improve when including CNVs into the model (BayesA+CNV, BayesB+CNV and GBLUP+CNV), but on average, MSE was higher for these models.

**Table 3:** Mean squared error (MSE) of genomic predictions of different models using all deletions and biallelic duplications (173 CNVs)

Trait	BayesA	BayesA+CNV	BayesB	BayesB+CNV	GBLUP	GBLUP+CNV
BW	0.87	0.86	0.89	0.85	0.88	0.89
CW	0.10	0.14	0.11	0.12	0.12	0.12
CY	0.18	0.21	0.20	0.23	0.19	0.21
MW	0.29	0.29	0.30	0.26	0.24	0.25
MY	0.28	0.32	0.26	0.22	0.27	0.22
PW	0.08	0.11	0.10	0.07	0.09	0.11
PWG	25.76	25.45	24.32	24.49	23.76	24.38
PY	0.16	0.16	0.14	0.15	0.15	0.11
WG	18.32	20.85	20.14	20.76	14.60	16.68

In order to measure the degree of inflation or deflation of direct genomic breeding values (DGV), the slope of the regression (b1) of dEBVs on DGV was evaluated. Table 4 shows the estimates of b1 for all nine traits. Model BayesA and BayesA+CNV resulted in inflated estimates compared to the other models. On average GBLUP performed the best in terms of scale.

**Table 4:** Inflation estimates (b1) of genomic prediction of 9 traits using different models using all deletions and biallelic duplications (173 CNVs)

	b1(dEBV,DGV)					
Trait	BayesA	BayesA+CNV	BayesB	BayesB+CNV	GBLUP	GBLUP+CNV
BW	1.06	0.96	0.78	0.95	1.04	0.93
CW	1.78	1.52	1.19	1.23	0.90	1.09
CY	1.82	1.74	1.44	1.31	1.12	1.18
MW	1.56	1.39	1.10	0.90	0.94	0.96
MY	1.28	1.33	1.15	1.09	1.02	1.15
PW	1.37	1.41	1.21	1.12	1.12	1.22
PWG	0.84	0.91	0.90	0.88	0.89	0.92
PY	1.24	1.19	1.23	1.14	1.11	1.09
WG	0.83	0.92	0.86	0.82	0.90	0.92

A study using the same dataset [44] revealed genetic stratification among the samples. Population stratification could potentially affect the resulting genomic prediction accuracies; however a random cross-validation approach was adopted in this study so that the impact of stratification was minimized [45]. In general, the prediction accuracy of the DGV for most traits using only SNP was in concordance with the results reported in literature for Nellore cattle breed [37]. For example the prediction accuracy for BW using GBLUP was 0.24 as reported in [37], and it resulted in an accuracy of 0.20 here (Table 2). Additionally, although genomic prediction accuracies were computed using dEBVs as the response variable; the results shouldn’t greatly change if other unbiased measures of true genetic value (e.g., average corrected performances (YD) or DYD for bulls) instead of dEBVs were used.

## Conclusions

In this study, including copy number variation information into genomic selection proved to be beneficial for some traits. However, their impact varied from model to model and from trait to trait and a universal model is yet to be developed. The small increase in prediction accuracy seen when integrating CNVs could be due to their function either as causal genes or as tagging markers. This might help in the prediction of complex traits and explain part of the missing heritability that SNP markers fail to capture. Future efforts are warranted to better utilize CNV information in genomic evaluation methods.

## Additional file

Additional file 1: **Table S1.** Detail information of the CNVs detected with high confidence. (XLSX 37 kb)
